# Spontaneous Generation of Infectious Prion Disease in Transgenic Mice

**DOI:** 10.3201/eid1912.130106

**Published:** 2013-12

**Authors:** Juan-María Torres, Joaquín Castilla, Belén Pintado, Alfonso Gutiérrez-Adan, Olivier Andréoletti, Patricia Aguilar-Calvo, Ana-Isabel Arroba, Beatriz Parra-Arrondo, Isidro Ferrer, Jorge Manzanares, Juan-Carlos Espinosa

**Affiliations:** Instituto Nacional de Investigación y Tecnología Agraria y Alimentaria, Madrid, Spain (J.-M. Torres, J. Castilla, B. Pintado, A. Gutiérrez-Adán P. Aguilar-Calvo, A.-I. Arroba, B. Parra-Arrondo, J.-C. Espinosa);; Basque Foundation for Science, Bilbao, Spain (J. Castilla);; Ecole Nationale Vétérinaire de Toulouse, Toulouse, France (O. Andréoletti);; Hospitalet de Llobregat, Barcelona, Spain (I. Ferrer);; Universidad Miguel Hernandez, Sant Joan d´Alacant, Spain (J. Manzanares)

**Keywords:** prions and related diseases, spontaneous generation, transmissible spongiform encephalopathy, TSE, bovine spongiform encephalopathy, BSE, transgenic mice, bovine prion protein, bovine PrP, Gerstmann–Sträussler–Scheinker syndrome

## Abstract

We generated transgenic mice expressing bovine cellular prion protein (PrP^C^) with a leucine substitution at codon 113 (*113L*). This protein is homologous to human protein with mutation *102L*, and its genetic link with Gerstmann–Sträussler–Scheinker syndrome has been established. This mutation in bovine PrP^C^ causes a fully penetrant, lethal, spongiform encephalopathy. This genetic disease was transmitted by intracerebral inoculation of brain homogenate from ill mice expressing mutant bovine PrP to mice expressing wild-type bovine PrP, which indicated de novo generation of infectious prions. Our findings demonstrate that a single amino acid change in the PrP^C^ sequence can induce spontaneous generation of an infectious prion disease that differs from all others identified in hosts expressing the same PrP^C^ sequence. These observations support the view that a variety of infectious prion strains might spontaneously emerge in hosts displaying random genetic PrP^C^ mutations.

Transmissible spongiform encephalopathies (TSEs) are fatal neurodegenerative diseases that affect humans and animals and involve pathologic conversion of host cellular prion protein (PrP^C^) to a disease-related isoform (PrP^Sc^), as proposed in the protein-only hypothesis (*1*). Depending on how these encephalopathies originate, TSEs are classified as sporadic, genetic, or infectious. Most have been experimentally transmitted and, with some exceptions, the presence of PrP resistant to proteinase K digestion (PrP^res^) is related to their infectivity (*2**–**4*).

Human genetic TSEs are caused by >30 autosomal-dominant point mutations in the human prion protein gene (*Prnp*) and have been classified as Gerstmann–Sträussler–Scheinker syndrome, familial Creutzfeldt–Jakob disease, or fatal familial insomnia (FFI), according to the clinical symptoms. Some of these genetic diseases have been transmitted to primates or rodents, although transmission rates were low in most instances (*5**–**8*). Regarding TSEs, pathogenic mutations in *Prnp* are believed to predispose mutant PrP^C^ to convert spontaneously to a pathogenic isoform (*9**–**11*).

Several transgenic mouse models confirmed that PrP^C^ with mutations induces a spectrum of neurologic diseases with clinical or histologic features of TSEs (*12**–**15*). However, the crucial prediction that a disease-associated PrP mutation can spontaneously generate infectivity has only been demonstrated in mice carrying the mutation D177N, the mouse equivalent of the mutation associated with human FFI (*16*). Spontaneous appearance of infectivity has also been reported in transgenic mice expressing a mouse PrP^C^ with 2 point mutations (170N and 174T) that subtly affect the structure of its globular domain (*17*).

The first described and most common Gerstmann–Sträussler–Scheinker syndrome mutation causing ataxia is P*102L* (*18**,**19*). Bovine P*113L*, which has a leucine substitution at codon 113, is homologous to human P*102L* and mouse P*101L*. Although bovine PrP^C^ with the *113L* mutation has not been found in nature, it would be useful to establish whether this mutation could induce spontaneous generation of an infectious prion disease in a bovine PrP context. In this study, we analyzed the phenotype of transgenic mice expressing mutant *113L* bovine prion protein (BoPrP) and the ability of these mice to generate de novo infectious prions in comparison with control mice expressing the wild-type protein.

## Materials and Methods

### Ethics

Animal experiments were conducted in strict accordance with recommendations in the guidelines of the Code for Methods and Welfare Considerations in Behavioral Research with Animals (Directive 86/609EC). All efforts were made to minimize detrimental effects on animals. Experiments were approved by the Committee on the Ethics of Animal Experiments of the INIA Institute (permit no. CEEA2009/004).

### Transgenic Mice

The open reading frame (ORF) of the bovine *Prnp* gene was isolated by PCR amplification and cloned in a pGEM-T vector as described (*20*). The ORF was mutated by using the QuikChange II-XL Kit (Stratagene, La Jolla, CA, USA) with specific oligonucleotides (5′-CGGTCAGTGGAACAAGCTCAGTAAGCCGAAAACC-3′ and 5′-GGTTTTCGGCTTACTGAGCTTGTTCCACTGACCG-3′) according to procedures of the manufacturer. The P*113L*-PrP ORF was excised from the cloning vector by using restriction enzyme *Xho*I and inserted into MoPrP.Xho vector (*21*), which was also digested with *Xho*I. This vector contains the murine PrP promoter and exon-1, intron-1, exon-2, and 3′-untranslated sequences. Transgenic mice were generated by microinjection of DNA according to a published procedure (*22*).

### Neuropathologic Studies in Spontaneously Diseased *113L*BoPrP-Tg Mice

Brains were rapidly harvested from the skulls and fixed in 4% paraformaldehyde in phosphate buffer. Coronal slabs were embedded in paraffin and 5-μm sections of cerebrum, cerebellum, brain stem, and spinal cord were obtained by using a sliding microtome. De-waxed sections were stained with hemotoxylin and eosin, Congo red, or thioflavin, or processed for immunohistochemical analysis. Immunohistochemical analysis for detection of glial fibrillary acidic protein (GFAP) and cleaved caspase-3 was conducted by using a modified labeled streptavidin technique (LSAB2-System peroxidase; Dako, Glostrup, Denmark). The rabbit polyclonal antibody to GFAP (Dako) was used at a dilution of 1:600. Cleaved caspase-3 rabbit polyclonal antibody (D175, cell signaling) was used at a dilution of 1:50. Microglial cells were stained with the biotinylated lectin from *Lycopericon esculentum* (L-0651; Sigma, St. Louis, MO, USA) and used at a dilution of 1:100. PrP was immunolabeled with monoclonal antibody (mAb) 6H4 (Prionics, Schlieren, Switzerland) and used at a dilution of 1:30 in sections pretreated with 35% HCl for 2 min at 100°C and then with 96% formic acid for 10 min at room temperature. These procedures have been detailed elsewhere (*23*).

### Mouse Transmission Studies

For transmission studies, we used the Tg110 mouse line (*20*) that expresses wild-type bovine-PrP^C^ in a mouse *Prnp* null background. Inocula were prepared from brain tissues as 10% (wt/vol) homogenates. Individually identified 6–10-week-old mice were anesthetized and inoculated with 2 mg of 10% brain homogenate in the right parietal lobe by using a 25-gauge disposable hypodermic needle. Mice were observed daily and neurologic status was assessed weekly. When progression of TSE was evident or evident at 650 days postinoculation (dpi), animals were euthanized for ethical reasons. Once mice were euthanized, brains were collected, frozen, and analyzed by Western blotting. Samples fixed in buffered 10% formalin underwent histologic analysis, immunohistochemical analysis, or histoblotting. Spleens were frozen for Western blot analysis.

### Western Blot Analyses of PrP^res^

Frozen mouse brain samples were prepared as 10% (wt/vol) homogenates in 5% glucose in distilled water in grinding tubes (Bio-Rad, Hercules, CA, USA) by using a TeSeE Precess 48 homogenizer (Bio-Rad) following the manufacturer’s instructions. Samples were analyzed by Western blotting as described (*24*). For immunoblotting experiments, mAbs Sha31 (*25*), 9A2 (*26*), 12B2 (*26*), Saf84 (*25*), and R145 (Vetyerinary Laboratories Agency, Weybridge, UK) were used at concentrations of 1 μg/mL. Sha31 recognizes _156_YEDRYYRE_163_ epitope, 9A2 recognizes _110_WNK_112_ epitope, 12B2 recognizes _101_WGQGG_105_ epitope, Saf84 recognizes _175_RPVDQY_180_ epitope, and R145 recognizes _231_RESQUA_235_ epitope of the bovine PrP sequence.

### Histopathologic Analysis

All procedures involving brains from infected mice were performed as described (*27*). Samples were fixed in neutral-buffered 10% formalin (4% formaldehyde) before being embedded in paraffin. Once deparaffinated, 2-µm tissue sections were stained with hematoxylin and eosin. Lesion profiles of brains were established according to the standard method described by Fraser and Dickinson (*28*). For paraffin-embedded tissue blots, the protocol described by Andréoletti et al. (*29*) was used.

## Results

### Expression of *113L*BoPrP in Transgenic Mice

**Figure 1:**
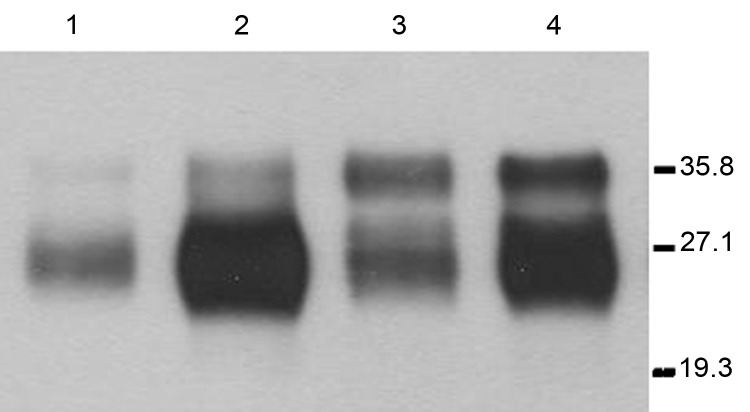
Immunoblots of brain extracts from hemizygous *113L*BoPrP-Tg037*^+/−^ 113L*BoPrP-Tg009*^+/−^* mouse lines compared with those of cow brain extract and BoPrP-Tg110 mouse brain extract. Brain homogenates were analyzed by Western blotting with monoclonal antibody 2A11 (*30*). Lane 1, *113L*BoPrPTg-009; lane 2, *113L*BoPrPTg-037; lane 3, Cow; lane 4, BoPrPTg-110. Equivalent amounts of total protein were loaded into each lane. *113L*, leucine substitution at codon 113; BoPrP, bovine prion protein. Values on the right are molecular masses in kilodaltons.

Seven lines (founders) of *113L*BoPrP^C^ heterozygous transgenic mice carrying the endogenous murine *Prnp* gene (*Prnp* mu^+/−^ *113L*bo^+/−^) were obtained. Lines *113L*BoPrP-Tg037 and *113L*BoPrP-Tg009 were selected on the basis of their expression levels, and bred to homozygosity in a murine *Prnp* null background. To achieve this expression, selected lines were crossed with *Prnp* null mice (*Prnp* mu^−/−^) to achieve transgene-hemizygous lines (*Prnp* mu^−/−^ *113L*bo^+/−^). Absence of the murine *Prnp* gene was determined by using PCR with specific primers. Transgene expression levels were then determined in brain homogenates by serial dilution and compared with PrP^C^ levels found in bovine brain homogenates. Transgene expression levels for the two 1-month-old mice with hemizygous Tg lines *113L*BoPrP-Tg037 and *113L*BoPrP-Tg009 were found to be ≈3× and 0.5×, respectively. Mutant *113L*BoPrP expressed in 009 and 037 transgenic lines showed an electrophoretic profile similar to that of wild-type bovine PrP^C^ from BoPrP-Tg110 mice or cow brain, although only small differences in glycoform ratios were observed (Figure 1). Next, by crossing hemizygous animals, we obtained transgene-homozygous animals (*Prnp* mu^−/−^ *113L*bo^+/+^) (*30*).

### Spontaneous Neurologic Disease in Transgenic Mice Expressing Mutant *113L*BoPrP

Spontaneous neurologic disease developed in *113L*BoPrP-Tg037 mice expressing mutant *113L*BoPrP. These mice had reduced lifespans compared with either non-Tg (*Prnp*^−/−^) mice or transgenic mice expressing similar or higher levels of wild-type BoPrP (Table 1). However, disease did not develop in *113L*BoPrP-Tg009 mice expressing low levels of mutant protein, and these mice had survival times similar to non-Tg (*Prnp*^−/−^) mice or BoPrP-Tg110 mice. Onset of clinical signs and survival times were dependent on the expression level of *113L*BoPrP (Table 1) (i.e., transgene-homozygous *113L*BoPrP-Tg037 mice showed an earlier onset of clinical signs and reduced survival times than hemizygous mice of the same line). Neurologic alterations generally involved motor impairment with ataxia affecting mainly the hind limbs. Mice showed a rough coat and prominent hunch at the early stages of clinical signs. Most mice had a wobbling gait and slight paralysis in the back limbs. Some mice had conjunctivitis and showed compulsive scratching in the head area. At the end stage of the disease, mice had highly restricted movement and lethargy. No signs of hyperactivity were detected in these mice.

**Table 1:** Onset of clinical signs and survival times for transgenic mice expressing different levels of mutant *113L*BoPrP or wild-type BoPrP*

Transgenic mouse line	Transgene expression level†	Onset of clinical signs, days ± SEM (no. diseased/no. tested)	Death, days ± SEM
*113L*BoPrP-Tg009^+/–^	0.5×	>550 (0/9)	>550
*113L*BoPrP-Tg009^+/+^	1×	>550 (0/10)	>550
*113L*BoPrP-Tg037^+/–^	3×	272 ± 33 (10/10)	345 ± 49
*113L*BoPrP-Tg037^+/+^	6×	187 ± 18 (6/6)	223 ± 47
BoPrP-Tg110^+/–^‡	4×	>550 (0/6)	>550
BoPrP-Tg110^+/+^‡	8×	>550 (0/10)	>550
Non-Tg (*Prnp*^–/–^)	0×	>550 (0/9)	>550

*BoPrP, bovine prion protein; *113L*, leucine substitution at codon 113; ^+/–^, hemizygous for bovine prion protein (*Prnp*) gene; ^+/+^, homozygous for bovine *Prnp* gene. All transgenic animals were murine *Prnp^−/−^*. †Relative to cattle PrP expression. ‡This BoPrP transgenic mouse line has been described (*20**,**22*).

### Neuropathologic Alterations in Transgenic Mice Expressing Mutant *113L*BoPrP

**Figure 2:**
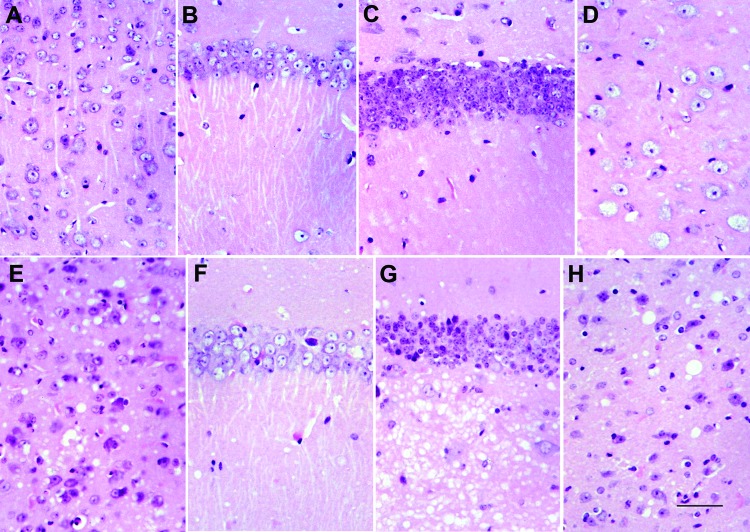
Comparison between homozygous bovine prion protein (BoPrP)-Tg110^+/+^ control mice (panels A–D) and hemizygous *113L*BoPrP-Tg037^+/−^ mice with end-stage disease (panels E–H) in parietal cortex (panels A and E), CA1 region of the hipocampus (panels B and F), dentate gyrus (panels C and G), and medial thalamus (panels D and H). Severe spongiosis is seen in the cerebral cortex, hilus ofdentate gyrus, and medial thalamus, but not in the CA1 area of the hippocampus and granule cell layer of the dentate gyrus. *113L*, leucine substitution at codon 113. Paraffin-embedded sections were stained with hematoxylin and eosin. Scale bar in panel H = 25 μm.

All *113L*BoPrP-Tg037 mice at the terminal disease stage showed spongiosis in the cerebral cortex, thalamus, and hilus of the dentate gyrus, but not in the CA1 region of the hippocampus and granule cell layer of the dentate gyrus, compared with age-matched control BoPrP-Tg110 mice (Figure 2). Marked granule cell loss, spongiosis in the molecular layer, granule cell layer, subcortical white matter, and Bergmann glia hypertrophy and hyperplasia were also observed in these mice at the terminal disease stage. However, at the early stages of clinical signs, no spongiform changes were found, although neuronal loss was observed when *113L*BoPrP-Tg037 mice were compared with age-matched control BoPrP-Tg110 mice. These findings were particularly evident in the hippocampus proper (including hilus) and granular cell layer of the cerebellum.

Changes were more pronounced in animals with severe clinical manifestations. Neurons with a shrunken cytoplasm and nucleus were observed in all *113L*BoPrP-Tg037 mice, and these appeared in the molecular layer of the cerebellum, hippocampus (mainly plexiform layers and hilus), thalamus, and pons. Morphologic PrP aggregates, congophilic materials, or thioflavin-positive deposits were not detected in *113L*BoPrP-Tg037 mice. Astrocyte gliosis was observed throughout the brain in *113L*BoPrP-Tg037 mice, even at the early stage of the disease. The number and size of reactive astrocytes increased, as shown by immunolabeling with antibody against GFAP, in the cerebral cortex, hippocampus, striatum, thalamus, cerebellum, and brain stem. Microglial proliferation, as visualized with *Lycopericum esculentum* lectin, was evident in brains of *113L*BoPrP-Tg037 mice but not in age-matched control mice.

### Modification of Biochemical Properties of Bovine-PrP^C^ by Mutation *113L*

**Figure 3:**
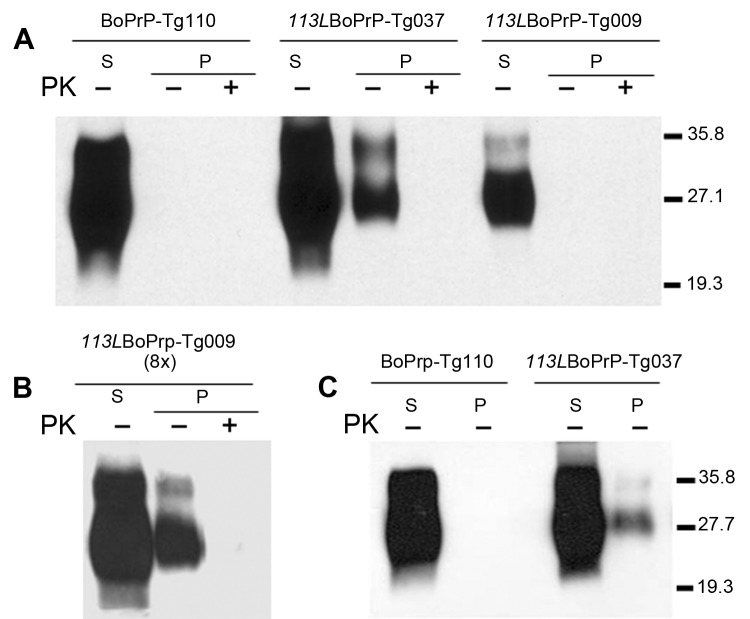
Host cellular prion protein (PrP^C^) solubility and proteinase K (PK) resistance studies in homozygous *113L*BoPrP-Tg037, *113L*BoPrP-Tg009, and control BoPrP-Tg110 mice. Western blot analysis with monoclonal antibody 2A11 of soluble (S) and insoluble (P) fractions obtained from mouse brain extracts (5% sarkosyl in phosphate-buffered saline, pH 7.4, previously cleared by centrifugation at 2,000 × *g*) after ultracentrifugation at 100,000 × *g* for 1 h. P fractions were treated with 5 μg/mL of PK (PK+) at 37°C for 60 min or left untreated (PK–). Panels A and B, show brain extracts from diseased *113L*BoPrP-Tg037 mice or from 500-day-old *113L*BoPrP-Tg009 and BoPrP-Tg110 mice. Panel C shows brain extracts from 30-day-old mice. In panels A and C, equivalent amounts of brain material were solubilized, centrifuged, and loaded onto the gel. In panel B, an 8-fold (8×) equivalent brain tissue mass was loaded to obtain equivalent PrP^C^ signals for the other 2 mouse lines. *113L*, leucine substitution at codon 113; BoPrP, bovine prion protein. Values on the right are molecular masses in kilodaltons.

To explore changes in biochemical properties of mutant *113L*BoPrP, we solubilized brain homogenates from control BoPrP-Tg110, *113L*BoPrP-Tg037, and *113L*BoPrP-Tg009 mouse lines in extraction buffer and ultracentrifuged them at 100,000 × *g* for 1 h. Western blotting of soluble and insoluble fractions indicated differential biochemical properties of mutant *113L*BoPrP compared with wild-type BoPrP (Figure 3); the *113L* mutation resulted in a more insoluble protein. This insolubility was detected in *113L*BoPrP-Tg037 and *113L*BoPrP-Tg009 mouse lines. However, when the *113L*BoPrP-Tg009 mouse line was compared with the other 2 mouse lines, the expression level was lower and insolubility was detectable only when an 8-fold equivalent brain tissue mass was used (Figure 3, panel B) to obtain equivalent PrP signal.

Insolubility was detected early in the lifespan (30 days after birth) of mice (Figure 3, panel C), which suggested that quantification of *113L*BoPrP would reflect a cumulative effect. PK resistance was not found in mutant *113L*BoPrP or in wild-type BoPrP, which were digested at the PK concentration used (Figure 3).

### Spontaneous Generation of Infectious Prions by *113L*BoPrP-Tg037 Mice

**Figure 4:**
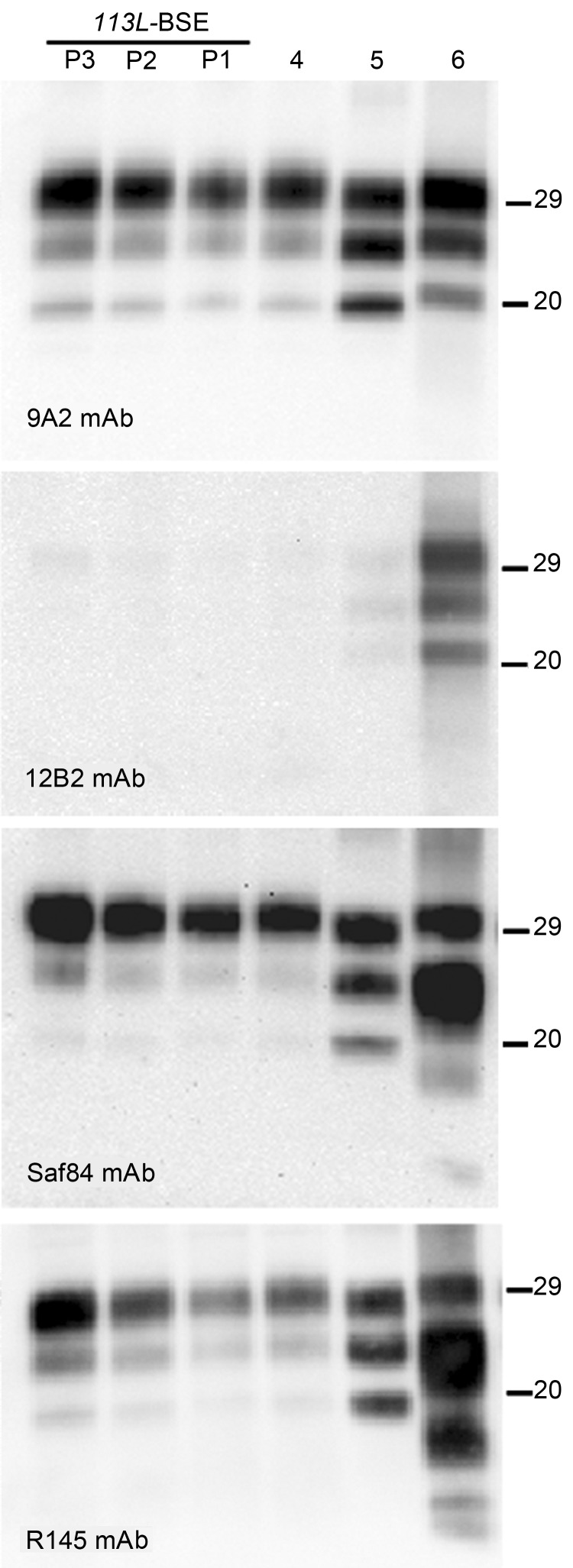
Comparative Western blot analyses of brain prion protein resistant to proteinase K digestion (PrP^res^) from BoPrP-Tg110 mice infected with bovine spongiform encephalopathy (BSE)-C, *113L*-BSE, BSE-L, and BSE-H prions. Mice infected with newly generated *113L*-BSE prion at first (P1), second (P2), and third (P3) passages are compared with mice infected with BSE-C (P1) (lane 4); BSE-L (P1) (lane 5); and BSE-H (P1) (lane 6) prions. Each panel was identified by using the monoclonal antibody (mAb) listed at the bottom left. The same quantities of PrP^res^ were loaded in all lanes. BoPrP, bovine prion protein; *113L*, leucine substitution at codon 113. Values on the right are molecular masses in kilodaltons.

To test potential infectivity of brains of mutant *113L*BoPrP-Tg037 mice, we intracerebrally inoculated brain homogenates from sick animals into transgenic mice expressing wild-type bovine PrP (BoPrP-Tg110). In the first experiment (Table 2), we used a brain homogenate from a unique terminally sick animal. In this instance, neurologic signs developed in only 1 of 5 (attack rate 20%) inoculated *Tg110* mice. This mouse died at 333 dpi and contained a considerable amount of PrP^res^ in its brain, as shown by Western blot (Figure 4).

**Table 2:** Infectivity of *113L*BoPrP-Tg037 mice brain homogenates in BoPrP-Tg110*

Inoculum	Mean ± SEM survival time, days (no. diseased/no. tested)†		
	First passage	Second passage	Third passage
*113L*BoPrP-Tg037 (179 d old)‡	330 (1/5)	272 ± 38 (6/6)	252 ± 23 (6/6)
*113L*BoPrP-Tg037-pool (150–250 d old)§	322, 406 (2/6)	291 ± 23 (6/6)	255 ± 14 (7/7)
wtBoPrP-Tg110-pool (500–600 d old)	>650 (0/12)	>650 (0/12)	NA
Normal mouse brain (500–600 d old)	>650 (0/12)	>650 (0/12)	NA
Classical BSE-C (VLAPG817/00) ¶	295 ± 12 (6/6)	265 ± 35 (6/6)	NA
Atypical BSE-H (CASE 03–2095)#	292 ± 5 (6/6)	296 ± 7 (6/6)	NA
Atypical BSE-L (02.2528)**	207 ± 7 (6/6)	199 ± 1 (6/6)	NA

*BoPrP, bovine prion protein; *113L*, leucine substitution at codon 113; wtBoPrP, wild-type bovine prion protein; NA, not available; BSE, bovine spongiform encephalopathy. †Intracerebral inoculation with 2 mg brain tissue equivalent in BoPrP-Tg110^+/+^ (8× expression level). ‡Brain homogenate from a unique terminally sick animal. §Brain homogenate from a pool of 5 terminally sick mice that died at 150–250 d of age. ¶Brainstem homogenate of 1 cow naturally infected with classical BSE (RQ 225:PG817/00) supplied by the Veterinary Laboratories Agency (New Haw,  #Data from Torres et al. (*31*). **Brainstem samples from naturally affected cows given a diagnosis of atypical BSE-L by the Agence Française de Sécurité Sanitaire des Aliments (Lyon, France).

When brain homogenate from this mouse was reinoculated into 6 *Tg110* mice (second passage), neurologic signs developed in all recipients, and these mice showed a shorter mean ± SEM incubation period (272 ± 38 dpi), which was suggestive of an increased infectious titer that was maintained in subsequent passages (Table 2). These results were confirmed in a second independent experiment with a brain homogenate derived from a pool of 5 terminally sick *113L*BoPrP-Tg037 mice. In this instance, 2 of 6 (attack rate 33%) inoculated mice were infected (*Tg110* mice) (33% attack rate) and had incubation periods of 322 and 406 dpi, respectively. As in the first experiment, second passage (using a pool containing both mouse brains) produced an attack rate of 100% and a shorter incubation period (291 ± 23 dpi), which were maintained in subsequent passages (Table 2).

Brain homogenate from *Tg110* mice expressing comparable amounts of wild-type BoPrP, as well as brain homogenate from healthy mice, was also used to inoculate uninfected mice, which served as negative controls. Neurologic signs did not develop in any of the inoculated mice (Table 2). These mice were euthanized at 650 dpi and did not show any PrP^res^ in their brains by Western blot.

For comparative studies, material from the brainstem of cows that contained classical bovine spongiform encephalopathy (BSE), atypical BSE-H, and atypical BSE-L prions was also inoculated into mice by the same procedure. These 3 inocula induced a typical neurologic disease after primary transmission and showed an attack rate of 100% (Table 2). Survivals times of mice inoculated with brainstem of *113L*BoPrP-Tg037 mice on second and third passages were similar to those produced by the classical BSE-C isolate (Table 2), as well as by other isolates reported for the same *Tg110* mouse line (*20**,**22**,**32*).

### Properties of P*113L-*BSE Prion

Western blot analysis with mAb 9A2 against brain-PrP^res^ produced by BoPrP-Tg110 mice infected with the new *113L-*BSE prion (Figure 4, panel A) showed a typical BSE PrP banding pattern characterized by small fragments (19-kDa fragment for the aglycosyl band) and prominent diglycosylated species in all challenged PrP^res^-positive mice. This result was indistinguishable from that produced by classical BSE-C prion in these mice but differed from that observed after inoculation with atypical BSE-H or BSE-L prions (Figure 4, panel A).

Further characterization of PrP^res^ with other mAbs showed that the new *113L-*BSE prion was not recognized by mAb 12B2 (Figure 4, panel B), whose epitope (_101_WGQGG_105_ according to the bovine PrP sequence) is known to be poorly protected from PK digestion (*26**,**32*) in the classical BSE-derived prion but well preserved in the atypical BSE-H prion (Figure 4, panel B) (*31*). Furthermore, PrP^res^ immunolabeling with mAbs Saf84 and R145 showed that mice infected with the new *113L*-BSE prion, in contrast to mice infected with the H-type prion, did not show the characteristic PrP^res^ band profile (4 bands) of cattle BSE-H, but showed a PrP^res^-profile (3 bands) similar to that of the BSE-C prion (Figure 4, panels C, D).

Comparative study of PrP^Sc^ accumulation in spleen from *Tg110*-infected mice showed that mice infected with *113L*-BSE or BSE-C prions consistently showed positive results for presence of PrP^res^ by Western blot. In contrast, no PrP^Sc^ deposits were detected in mice infected with either BSE-L or BSE-H prions. Similar results were obtained in subsequent passages.

**Figure 5:**
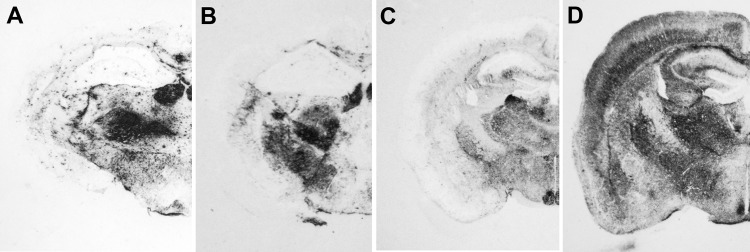
Immunochemical analysis of paraffin-embedded tissue blots of representative coronal sections of the hippocampus, showing deposition patterns of abnormal isoform of host-encoded prion protein in brains from BoPrP-Tg110 mice infected with bovine spongiform encephalopathy (BSE)-C (A), *113L*-BSE (B), BSE-H (C), and BSE-L (D) prions. BoPrP, bovine prion protein; *113L*, leucine substitution at codon 113. Monoclonal antibody Sha31 stained by using the procedure of Andréoletti et al. (*29*). Original magnifications ×20.

**Figure 6:**
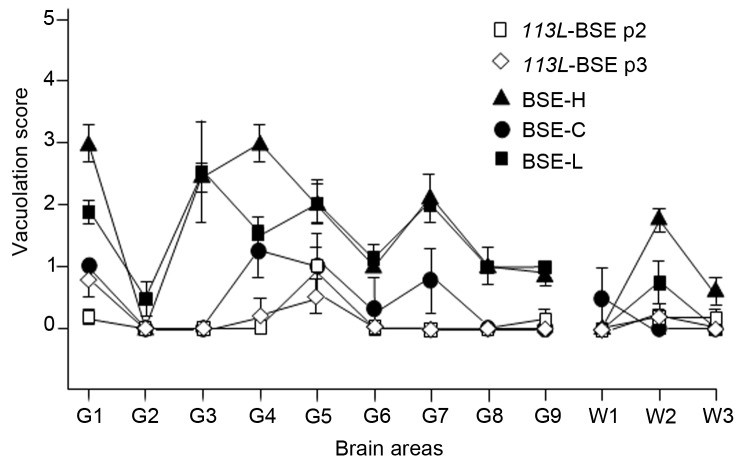
Vacuolar lesion profile in brains from BoPrP-Tg110 mice inoculated with bovine spongiform encephalopathy (BSE)-C (black circles, n = 6 animals), BSE-H (black triangles, n = 6 animals), BSE-L (black squares, n = 5 animals), *113L*-BSE second passage (black squares, n = 5 animals), and *113L*-BSE third passage (open diamonds, n = 5 animals) prions. Lesion scoring was conducted for 9 areas of gray matter (G) and 3 areas of white matter (W) in mouse brains. G1, dorsal medulla; G2, cerebellar cortex; G3, superior colliculus; G4, hypothalamus; G5, medial thalamus; G6, hippocampus; G7, septum; G8, medial cerebral cortex at the level of the thalamus; G9, medial cerebral cortex at the level of the septum (G9); W1, cerebellum; W2, mesencephalic tegmentum; W3, pyramidel tract. BoPrP, bovine prion protein; *113L*, leucine substitution at codon 113. Error bars indicate SE.

We next examined vacuolation and PrP^Sc^ distribution in the brain, which are known to vary by strains/TSE prions (*28**,**33*). In general, we observed that PrP^Sc^ deposition patterns in brains of *113L*-BSE–infected mice were different from mice infected with BSE-H or BSE-L prions, but these overlapped mostly with those infected with BSE-C prion (Figure 5). However, *113L*-BSE–infected mice at the terminal stage of disease showed only spongiform changes that remained limited to the thalamus even after 3 passages (Figure 6). This finding was in contrast with lesion profiles observed in the mice infected with BSE-C, BSE-H or BSE-L prions (Figure 6) in which substantial vacuolar changes were observed in various brain areas. These results indicate that *113L*-BSE is an authentic infectious prion that phenotypically differs from BSE-H and BSE-L prions but has biochemical characteristic and histopathologic features similar to those of the classical BSE-C prion.

## Discussion

We showed that the *113L* mutation in the bovine *Prnp* gene gives rise to a spontaneous neurodegenerative disease when expressed in transgenic mice. Neurologic symptoms of ataxia, rigidity, and lethargy accompanied by spongiform degeneration throughout the brain spontaneously develop in these mice. The rate at which illness progresses is related to expression levels of the mutant *113L*BoPrP (Table 1). Neurologic alterations did not develop in several mouse lines expressing similar or higher levels of wild-type bovine PrP^C^ during their lifespan, which is similar to observations in wild-type mice (Table 1) (*20*). Although the mechanism inducing the disease is unclear, we suggest that the *113L* mutation in bovine PrP^C^ could give rise to a different structure with respect to wild-type PrP^C^, which shows reduced solubility (Figure 3). Enhanced aggregation of mutant PrP could affect the appearance of the disease. Other mutations in the *Prnp* gene have also been related to enhanced aggregation of the mutant PrP in transgenic mice (*12**–**14**,**34*). However, the mechanisms through which these mutations may influence the aggregation properties of PrP^C^ are unclear.

In previous studies, overexpression of murine PrP carrying the *101L* mutation (equivalent to human *102L* and bovine *113L* mutations) led to spontaneous neurodegenerative disease in mice (*15**,**35**,**36*). However, when this mutation was introduced into the murine *Prnp* gene by gene targeting, mice homozygous for the *101*L mutation showed no spontaneous spongiform encephalopathy (*37*). As proposed by Manson et al., the lifespan of a mouse carrying only 1 or 2 copies of the mutant gene is insufficiently long enough to enable the stochastic event that makes TSE occur (*37*). Transgenic mice expressing high levels of human PrP^C^ carrying the familial *101L* mutation were reported to be free of disease (*38*). These results suggest that an equivalent mutation in PrPs from different species might have different structural consequences. A possible explanation is that species-specific interaction sites for PrP cofactors or chaperones are required, and that in mice they are compatible for bovine PrP but not for human PrP.

We also show that spontaneous neurodegenerative disease induced by the single *113L* amino acid substitution is transmissible to mice expressing wild-type bovine PrP^C^, indicating spontaneous generation of infectious prions. Transmissibility of this genetically initiated disease to mice not carrying *113L* mutations provides crucial support for a causal link between PrP misfolding and the spontaneous generation of a transmissible prion. Whether the small amount of insoluble PrP we detected in brain homogenates (Figure 3) constitutes the infectious prion in our mice, or some other as yet uncharacterized species, remains to be determined.

Several transgenic mouse models expressing PrP with various familial mutations have been reported (*12**–**14**,**34*).Most of these transgenic mouse models have confirmed that the presence of these mutations triggers spontaneous disease, but spontaneous generation of a transmissible prion has only been reported for mutation D178N, associated with human FFI (*16*). This study reported the spontaneous appearance of infectivity in knock-in mice carrying the mouse-equivalent D177N mutation. Spontaneous infectivity has also been reported in transgenic mice expressing a mouse PrP with 2 point mutations (170N and 174T), which subtly affect the structure of its globular domain (*17*).

The new *113L* BSE prion generated shares some phenotypic features with the classical BSE-C prion when inoculated in the same *Tg110* mouse line according to various criteria: 1) apparent molecular mass of PrP^res^, 2) PrP^res^ glycosylation pattern, 3) lack of immunoreactivity with mAb 12B2, 4) pattern of labeling with mAbs Saf84 and R145, 5) detectable PrP^res^ in spleens of infected animals, and 6) spatial distribution of PrP^res^ in brain. However, the vacuolation profile in brain was distinct from those of all known bovine prion strains (classical BSE-C, atypical BSE-H, and atypical BSE-L prions). These differences were maintained after subsequent passages, indicating that the novel prion, spontaneously produced by transgenic mice expressing mutant *113L*BoPrP, is distinct from all known bovine prion strains, although it shares many phenotypic features with the classical BSE-C prion.

These observations demonstrate that mutations in bovine PrP can result in spontaneous generation of infectious prion diseases and support the hypothesis of a genetic origin for the epidemic BSE prion. Different features exhibited by the new *113L-*BSE prion, compared with those of the classical BSE prion, suggest that if the origin of BSE was genetic, it is unlikely that the causal mutation would be related to the *113L* mutation. However, slight phenotypic differences observed could be the results of evolution of the epidemic BSE prion in field conditions in cattle, which must be different from those of our transgenic mouse model. Although BoPrP with the *113L* mutation has not been found in nature, a potential pathogenic mutation (E211K) within PrP has been recently reported in a cow with an H-type BSE phenotype (*39*). This mutation is equivalent to a common mutation (E200K) in humans, which is associated with genetic TSEs.

Spontaneous appearance of infectivity reported in transgenic mice expressing a mutated BoPrP and in mice expressing mutated mouse PrP reported by Stöhr et al. (*40*) supports the hypothesis that infectious TSE prions, could originate by a random genetic mutation that can induce de novo generation of infectious prions, and that this mechanism could constitute a source of prion diversity. These considerations enable us to hypothesize that the BSE epidemic could have begun by a random genetic mutation that was able to generate de novo infectious prions, which were included in meat and bone meal fed to cattle and then broadly expanded in the cattle population. According to this hypothesis, a key strategy for controlling BSE would involve preventing cows from consuming products from cows with spontaneous cases of BSE.
